# Understanding the Fertility Desires and Intentions among HIV-Positive Men Living in Ontario: Survey Instrument Development and Validation

**DOI:** 10.1177/2325958219831018

**Published:** 2019-02-25

**Authors:** Joseph Nguemo Djiometio, Tony Antoniou, Vicki Kristman, Rebecca Schiff, Molly Gamble, Logan Kennedy, Mark Yudin, Mona Loutfy

**Affiliations:** 1 Department of Health Sciences, Lakehead University, Thunder Bay, Ontario, Canada; 2 Women’s College Research Institute, Women’s College Hospital, University of Toronto, Toronto, Ontario, Canada; 3 Ryerson University, Toronto, Ontario, Canada; 4 Department of Family and Community Medicine, St Michael’s Hospital, Toronto, Ontario, Canada; 5 Institute for Work & Health, Toronto, Ontario, Canada; 6 Division of Human Sciences, Northern Ontario School of Medicine, Lakehead University, Thunder Bay, Ontario, Canada; 7 Dalla Lana School of Public Health, University of Toronto, Toronto, Ontario, Canada; 8 Department of Obstetrics and Gynecology, St Michael’s Hospital, Toronto, Ontario, Canada

**Keywords:** HIV/AIDS, fertility, survey, development, validation

## Abstract

Men with HIV have highlighted the importance of understanding their fertility desires. However, most research has focused on women. We aimed (1) to develop a survey instrument to assess fertility desires and intentions among HIV-positive men and (2) to assess its face, content, and construct validity, as well as test–retest reliability and internal consistency. Principal component analysis was used for construct validity analysis in a sample of 60 men with HIV. The test–retest reliability and internal consistency were assessed using Spearman correlation and Cronbach α, respectively. The initial and the final version of the questionnaire consisted of 10 domains and 14 constructs. We found a one-component model for the 3 constructs analyzed and Cronbach α values were ≥.70. Test–retest statistic was stable with Spearman correlation >0.70. In conclusion, a reliable and valid questionnaire was developed for determining the fertility desires and intentions of men with HIV.

**What Do We Already Know about This Topic?**

To date, other instruments have been designed to assess fertility needs exclusively for heterosexual and bisexual men living with HIV.

**How Does Your Research Contribute to the Field?**

This instrument will be used in future studies to determine the fertility desires and intentions of HIV-positive men regardless of their sexual orientation.

**What Are Your Research’s Implications toward Theory, Practice, or Policy?**

This instrument is needed to collect information that will allow clinicians, AIDS Service Organizations (ASOs), and the HIV-positive community to become more aware of the desires, intentions, and needs of men in regard to parenting.

## Introduction

Advances in HIV treatment, management, and support over the past 3 decades have contributed to tremendous shifts in the lives of people living with HIV.^1^ As people with HIV live longer, questions regarding the potential for long-term partnerships and/or having children have become increasingly important. Previous research has shown that fatherhood is a life-changing experience for the male population.^2^ However, HIV research concerning reproductive health and family planning to date has focused mainly on women.^3^ It is also evident that available studies on men have been disproportionately biased toward the perspective of heterosexual men.^4^ Studies from the United States, Europe, and Africa^5–8^ have postulated that a high proportion of men living with HIV in these countries want to have children. In addition, previous studies have identified several key factors influencing men living with HIV regarding their decision to have children. These factors include age, number of living children, health status, fear of discrimination, community pressure, and the attitudes of health care providers, as well as assistance in achieving pregnancy for men living with HIV and their partners.

While many variables influencing the desires and intentions of men living with HIV to pursue having children have been identified, many other variables or traits are equally important and also need to be considered.^9^ Each of these traits may influence whether men would contemplate parenting and warrant examination to determine how collectively and independently each factor might be associated with planning to raise children and to become a parent. Moreover, current instruments do not investigate the ultimate end point of a model: the actual behavior undertaken to achieve a pregnancy. Developing an instrument that includes all of these factors may help health care practitioners to provide better service and resources to men living with HIV. These data are fundamental in the development of services, support, and resources in relation to fertility and fatherhood for men who are HIV positive and want to have families in the future.^10^ Therefore, the objectives of this study were: (1) to develop a survey instrument to assess fertility desires and intentions among HIV-positive men and (2) to assess the face, content, and construct validity, as well as test–retest reliability and internal consistency of the developed instrument. We specifically determined the psychometric properties of the survey using scales measuring fertility feelings, desires, and intentions.

## Material and Methods

### Selection of Constructs and Items

Initial constructs and items were drawn from a previously validated survey instrument that assessed the fertility desires and intentions among Canadian HIV-positive women.^11^ This survey instrument consisted of 189 items.^11^ This survey was also developed using the Traits-Desires-Intentions-Behavior framework and consisted of 12 domains^11^:

1. Interest/desire to have children.
2. Intent to have children in the future.
3. Behavior related to the pursuit of fertility.
4. Menstrual, birth control, and sexual history.
5. Previous pregnancy and birth history.
6. Perceived support for becoming pregnant.
7. Satisfaction with providers.
8. Needs assessment.
9. HIV medical history.
10. Demographics.
11. Anxiety and depression.
12. HIV stigma.

Additional items were selected from a survey assessing reproductive views among HIV-positive heterosexual men in London, England, that consisted of 17 items.^10^ The survey examined parenting experience, attitudes toward parenthood, information needs in relation to reproductive support and service provision, decision-making, unprotected sex in relation to procreation, and the meaning of fatherhood to a person.^10^ The wording deemed most appropriate for selected items was used with minor revisions to tailor the survey instrument to the male and Canadian contexts.

### Content Validation

The content validation was carried out by 6 experts with knowledge in infectious diseases (2), HIV primary care (2), and internal medicine (2). Experts were members of the Interdisciplinary HIV Parenting Research and Exchange Group. Two rounds of content validation were undertaken to assess whether the content of the questionnaire was appropriate, improved upon, and relevant for the purposes of the study. Initially, 5 experts independently rated the relevance of each item using a 4-point Likert scale (1 = not relevant, 2 = somewhat relevant, 3 = relevant, and 4 = very relevant). The content validity index (CVI) was then computed after the relevance of each item was rated. The relevance of each item was dichotomized to not relevant (if item rated 1 or 2) and relevant (if item rated 3 or 4). In addition to collecting quantitative data, the experts were asked to provide comments on the relevance of each item that was rated as not relevant or somewhat relevant. This approach was particularly effective in rewording some of the items and identifying indicators of whether a particular construct was not well represented by the existing items or whether there were enough items to cover the objective of the study. Finally, the updated overall questionnaire was reviewed by a sixth expert who edited and rated the relevance of the items and the rewording of the items. The draft online instrument was then created using LimeSurvey (Hamburg, Germany).

### Face Validation

Face validation was conducted by 5 participants. Criteria for inclusion in face validation were HIV positive, 18 years or older, identify as male, and live in Toronto. Participants completed the online instrument, which had been revised through content validation, using the computer-assisted personal interviewing (CAPI) model. Participants answered questions on the following topics using the CAPI model:

- Demographics.
- Contraception and sexual history.
- Interest/desire to have children.
- Intent to have children in the future.
- Behavior(s) related to the pursuit of fertility.
- Conception and parenting.
- Perceived support for becoming a parent.
- Satisfaction with providers related to fertility goals.
- Needs assessment.
- HIV history.

These participants also took part in a focus group in order to provide their thoughts about the layout of the questionnaire in terms of the length, flow, clarity, and use of language. Focus group sessions also involved examination of the questionnaire content by discussing each of the 10 themes mentioned above. Following face validation, the psychometric properties of the survey were determined using scales measuring fertility feelings, desires, and intentions.

### Construct Validation

A sample of 60 men living with HIV aged ≥18 years were recruited for this pilot study from a large medical clinic in downtown Toronto. This clinic provides care to more than 2700 people living with HIV, most of whom are men. The study was carried out using a cross-sectional design. Participants completed the interview using a CAPI model. Computer-assisted personal interviewing interviewers enter the data directly into a LimeSurvey online database based on the answers of the participants. Assessment for construct validity was based on data from the content validation, face validation, and the pilot study.

### Internal Consistency and Reliability Assessment

Internal consistency of the instrument under development was assessed using Cronbach α. In addition, 20 participants who completed the pilot study were invited for retest at intervals of 2 to 5 weeks between tests, and a Spearman ρ correlation was computed to assess reliability.

### Analysis

The first criterion of content validity was a CVI ≥ 0.78. The second criterion was the comments of experts and, to some extent, the importance of the variable measured based on the survey assessing fertility intentions and desires among women living with HIV.^11^ The second criterion was applied to items with a CVI < 0.78. For face validity, the data collected from the focus group were transcribed using a verbatim style and analyzed using a direct content analysis. Principal component analysis (PCA) was used for construct validity analysis. Our sample did not reach the recommended minimum number of participants for factor analysis; therefore, we examined the Kaiser-Meyer-Olkin (KMO) and the Bartlett test of sphericity to ensure our sample met adequate requirements for analysis.^12^ The cutoff value for KMO was ≥0.50 and the Bartlett test of sphericity was significant at a value ≤0.05. A factor is reliable if it has 4 or more loadings of at least 0.40.^13^ Therefore, the criteria used to obtain the best fitting structure were as follows:

1. The factors highlighted by the rotation were selected according to the eigenvalue >1 for each factor.^12^
2. Items with factor loading <0.40 were removed.
3. Subfactors with less than 4 variables were considered for combination into one concept.

Cronbach α was used to examine the internal consistency of the structure of the constructs in an iterative process in order to identify items that did not contribute or had contributed negatively to the Cronbach α of the construct. Therefore, items with a negative impact were subsequently removed. The cutoff value for Cronbach α for the developed instrument was .70 and indicated a good correlation.^14^ A Spearman rank-order correlation (ρ) was run to determine the relationship between test and retest for the 3 selected items that directly measured fertility desires and intentions. The study sought evidence of a correlation of at least 0.70 for the test–retest reliability.^15^

## Results

### Domains and Constructs of the Survey

The specific domains and constructs identified are specified in Table 1. The draft questionnaire consisted of 10 domains and 14 constructs.

**Table 1.** Draft Questionnaire Domains and Constructs.

Domain	Construct
Demographics	Demographics
Contraception and sexual history	Contraception and sexual history
Interest/desire to have children	1. Feelings 2. Desires
Intent to have children in the future	1. Plans to have children 2. Worries
Behavior(s) related to the pursuit of fertility	Behavior(s) related to the pursuit of fertility
Conception and parenting	Conception and parenting
Perceived support for becoming a parent	1. Support 2. Fear 3. Experience with fear
Satisfaction with providers related to fertility goals	Satisfaction with providers related to fertility goals
Needs assessment	Needs assessment
HIV history	HIV history

### Content Validity

The draft questionnaire submitted to experts comprised of 122 items, which included dichotomous, multiple-choice, and Likert-type questions. Following the CVI calculation, items that were deemed irrelevant (CVI ≤ 0.78) were deleted or edited or retained for a reason. As depicted in Table 2, a total of 15 items were deemed to be invalid: 7 were deleted, 2 were revised, and 6 were kept in their original format because they were used to measure important determinants of fertility, such as the capability for fathering and the attitude of health care providers about fathering for men living with HIV. All of the remaining items were deemed valid, with CVIs ranging from 0.80 to 1.00.

**Table 2.** Content Validity Index (CVI) <0.78 of the Items and the Action Taken.

Construct	Item	CVI	Action Taken
Feelings	1. I feel that being a father would increase my self-esteem.	0.6	Edited
	2. I would like having children but have been unable.	0.6	Deleted
Intent to have children in the future	1. Because HIV medications will let me live longer, I am considering becoming a father.	0.6	No action
	2. As a man with HIV, I can go to a fertility clinic and access medical help to have a child, including egg donor and surrogates.	0.6	Deleted
Worries	I am worried that if I become a father, my child will be born HIV positive.	0.6	No action
Contraception and sexual history	1. Have you had sexual intercourse (vaginal) with a female partner EVER in your lifetime?	0.6	No action (skip pattern recommended)
	2. If you have any children, has your doctor asked you about the HIV status of the person who carried that pregnancy?	0.4	Deleted
Fertility and parenting history	1. If you have biological children, what is your current relationship with them?	0.6	Deleted
	2. If you have biological children, have any of these children been diagnosed with HIV?	0.6	Deleted
	3. How many children LIVE with you now? (Include children that you care for but did not give birth to)	0.6	Deleted
	4. If you have children that you are caring for, how old are they? (please list their ages)	0.6	Deleted
Satisfaction with providers related to fertility goals	Do you have a family doctor?	0.6	No action
	I can trust my family doctor.	0.6	No action
HIV history	1. Do you currently have any of the following sexually transmitted infections?	0.6	Edited
	2. Are you currently taking hepatitis C treatment? (This medication should not be taken when trying to have a child.)	0.6	No action

The experts suggested the addition of 4 new items:

1. I am happy with my life without children.
2. I would like to become a father without parenting the child(ren).
3. I am willing to pay for fertility clinic resources so as to protect woman from HIV transmission.
4. If I were to become a father, I would be worried that my children will experience discrimination at school.

Finally, the last expert reviewed the instrument and no further changes were recommended. In addition, the expert suggested providing participants with the option of completing sensitive questions alone, in the absence of an interviewer. Furthermore, the web survey was developed with some screening/logic checks built-in for heteronormative items.

### Face Validity

Five men living with HIV, including 2 heterosexual men and 3 nonheterosexual men (ie, gay or bisexual men), were involved in the face validity process. The 3 nonheterosexual men were Caucasian and born in Canada. The 2 heterosexual men were immigrants from Black and Hispanic backgrounds. Participants were aged between 22 and 57 years; the median and mean ages being 44 and 43 years, respectively. All participants were currently on antiretroviral medication. The CD4 counts of all participants were >200 cells/mm^3^, and their viral loads were undetectable (<50 copies/mL). Participants indicated that the layout was adequate and that the time to complete the survey (25-30 minutes) was appropriate. Some participants pointed out that they were presented with questions that were not relevant to their experience. In these cases, they suggested adding a “not applicable” category, so that participants would not be forced to choose an answer they were not comfortable picking. Some participants found many questions or constructs (feelings, desires, and intention) were confusing, and they suggested that a definition of the questions or constructs would be helpful. Many of the participants also suggested rewording, rephrasing, and editing many items so that they were more clear. Most participants felt that the key aspects of fertility intentions and desires were well reflected in the survey and found the study very relevant and important. However, some participants suggested adding more questions to allow more choices for participants. Overall, in response to focus group feedback, definition and clarification of some constructs and items were made in the draft web instrument and 19 items were added to the questionnaire (Table 3).

**Table 3.** Face Validation: New Items Suggested by Community Members.

Construct	Items Added
Sociodemographics	Who do you live with?
Desires	1. Being diagnosed with HIV affects my decisions about not becoming a father.
	2. If I was in a situation where I could become a father, I would not want that to happen.
Worries	1. I would be worried that my HIV medications will affect my ability to care about my child.
	2. I would be worried that if I have children I would not focus enough on my medication.
Behavior(s) related to the pursuit of fertility	1. I have spoken to my surrogate mother or co-parent about having a baby.
	2. I have spoken to a community member about having a baby.
Experience with conception/parenting	1. How many children have you ever parented?
	2. How many children have you ever parented since you were diagnosed with HIV?
Support	1. My community wants me to have a child.
	2. By having a child, it would make my community happy.
Fear	I am afraid of being judged negatively by my other child(ren) of trying to have more children.
Satisfaction with providers related to fertility goals	1. I am comfortable sharing my concerns about becoming a father with my case worker/social worker.
	2. I am comfortable talking to my case worker/social worker about fatherhood as a man living with HIV.
	3. I can trust my case worker/social worker.
	4. I am satisfied with the service I receive from my case worker/social worker.
	5. I am satisfied with the amount of parenting planning information I received from my case worker/social worker.
	6. Has your case worker/social worker talked to you about fatherhood?
	7. Has your case worker/social worker ever advised you against having children?

### Pilot Testing

Participants were recruited in March to April 2016 from an HIV clinic in Toronto. The majority (78.3%) was identified as nonheterosexual. The age of the participants ranged from 26 to 57 years, with a median age of 44 years (interquartile range [IQR]: 29-56) and a mean of 43.3 years (standard deviation: 10.4). The majority (n = 32; 53.3%) reported an annual income of more than $40,000 per year. The time since diagnosis with HIV ranged from 0 to 33 years, with a median time of 11 years (IQR: 4-18). The most common mode of HIV acquisition among participants was sex with a male partner (70%). The CD4 counts of all participants were ≥200 cells/mm^3^, 99% reported undetectable (<50 copies/mL) viral loads, and 98% of participants were currently on antiretroviral treatment. Approximately 28% of the participants had parented at least 1 child and 20% had parented at least 1 child since being diagnosed with HIV.

### Principal Component Analysis and Internal Consistency Reliability

The KMO coefficient for the data set “feelings” was 0.81 and the Bartlett test of sphericity was statistically significant (χ^2^ = 133.77, *df* = 6, *P* < .001), indicating a positive fit for factor analysis. Analysis of the variation showed 1 factor recording an eigenvalue above 1 that explained 74.44% of variance. The factor loadings varied between 0.78 and 0.92 and Cronbach α computed for the internal consistency reliability of the total subscale was .88 (Table 4). Therefore, the item-total statistics suggested that all items can function well as a single concept.

**Table 4.** Component Matrix and Internal Consistency Reliability for “Feelings.”

	Factor Loadings	Cronbach α
I think that being a father would increase my worth in life	0.92	.88
I think I would feel fulfilled by caring for children	0.9	
I think that being a father is important to me	0.85	
I think children give meaning to life	0.78	

The KMO coefficient for the data set “interest/desire to have children” was 0.66 and the Bartlett test of sphericity was statistically significant (χ^2^ = 233.14, *df* = 36, *P* < .001), indicating a positive fit for factor analysis. Analysis of the variation showed 2 factors recording an eigenvalue above 1 that explains 61.69% of variance. The PCA confirmed 2 subfactors. Three items (factor loadings: 0.76-0.93) loaded on the first subfactor and 5 items (factor loadings: 0.32-0.82) loaded on the second subfactor. Cronbach α was computed for the internal consistency reliability of the construct (α = .47).

The item “If I was in a situation where I could become a father, I would not want that to happen” reported a negative factor loading and was subsequently removed. In addition, the factor loading of the item “I am happy with my life without children” (0.32) was below our cutoff value and the item was subsequently removed (Table 5). When the 2 items were removed, the resulting single structure including 7 items showed a final internal consistency reliability of 0.74.

**Table 5.** Rotated Component Matrix and Internal Consistency Reliability for “Interest/Desire to Have Children.”

	Factor Loadings		Cronbach α If Items Deleted
	Subfactor 1	Subfactor 2	
I would like to become a father in the future	0.93	0.02	.44
I have thought about becoming a father in the future	0.80	0.08	.42
If I was in a situation where I could become a father, I would want that to happen	0.76	0.34	.37
If I was in a situation where I could become a father, I would not want that to happen	−0.74	−0.08	.62
I am happy with my life without children	−0.66	0.32	.57
Being diagnosed with HIV affects my decisions about not becoming a father	−0.10	0.82	.33
I would like to become a father without parenting the child(ren)	−0.20	0.75	.4
Available fertility technologies and options for people living with HIV affect my decisions about becoming a father	0.29	0.71	.29
Being diagnosed with HIV affects my decisions about becoming a father	0.30	0.57	.25

The KMO coefficient for the data set “intent to have children in the future” was 0.64 and the Bartlett test of sphericity was statistically significant (χ^2^= 57.39, *df* = 10, *P* = .00), indicating a positive fit for factor analysis. Analysis of the variation in the construct revealed 1 factor with an eigenvalue above 1, explaining the 45.09% variance. The PCA confirmed a single-concept structure where the factor loadings varied between 0.47 and 0.83. Cronbach α computed for the internal consistency reliability of the construct (α = .68) was below our cutoff value. Therefore, the item “I would be willing to consider adoption as alternative to having a biological child” was deleted and Cronbach α was .70 (Table 6).

**Table 6.** Component Matrix and Internal Consistency Reliability for “Intent to Have Children in the Future.”

	Factor Loading	Cronbach α If Items Deleted
I am open to the idea of using medical techniques to help me become a father	0.83	.53
I am willing to pay for fertility clinic resources to become a father	0.81	.56
Because HIV medications will let me live longer, I am considering becoming a father	0.67	.63
As a man with HIV, I can have an HIV-negative child	0.49	.68
I would be willing to consider adoption as an alternative to having a biological child	0.47	.70

### Internal Consistency Reliability

The internal consistency of selected constructs indicated Cronbach α ≥ .70, which indicated that the questionnaire was consistently reliable (Table 7).

**Table 7.** Cronbach α of Selected Constructs.

	Number of Items Deleted	Current Number of Items	Cronbach α
Feelings	0	4	.88
Desires	2	7	.74
Intentions	1	4	.70
Worries	0	11	.84
Behavior(s) related to the pursuit of fertility	0	9	.71
Conception and parenting	0	6	.70
Support for becoming a parent	0	6	.86
Fear	1	6	.76
Experience with fear	0	5	.74
Satisfaction with providers related to fertility goals	0	5	.78

### Test–Retest Reliability

Table 8 provides an overview of the stability in the answers of respondents between the 2 tests with a Spearman ρ correlation varying between 0.71 and 0.85 for 3 selected items.

**Table 8.** Test–Retest Results using Spearman ρ Nonparametric Test Correlation.

	Test, n (%)	Retest, n (%)	Spearman *ρ*	*P* Value (Significance 2 tailed)
I have think about becoming a father in the future			0.71	.00
Strongly disagree	0 (0.0)	2 (10.0)		
Disagree	9 (45.0)	4 (20.0)		
Neither disagree nor agree	0 (0.0)	2 (10.0)		
Agree	6 (30.0)	7 (35.0)		
Strongly agree	5 (25.0)	5 (25.0)		
I would like to become a father in the future			0.83	.00
Strongly disagree	1 (5.0)	3 (15.0)		
Disagree	8 (40.0)	6 (30.0)		
Neither disagree nor agree	3 (15.)	2 (10.0)		
Agree	3 (15.0)	4 (20.0)		
Strongly agree	5 (25.0)	5 (25.0)		
Because HIV medications will let me live longer, I am considering becoming a father			0.85	.00
Strongly disagree	0 (0.0)	2 (10.0)		
Disagree	8 (40.0)	7 (35.0)		
Neither disagree nor agree	0 (0.0)	1 (5.0)		
Agree	8 (40.0)	7 (35.0)		
Strongly agree	4 (20.0)	3 (15.0)		

## Discussion

In this study, we created and validated an instrument specific to the measurement of motivations, fertility desires, and intentions, as well as actions taken to pursue fathering children for men living with HIV, regardless of their sexual orientation. Prior to this, there has been a lack of such an instrument. We not only developed a survey instrument to assess fertility desires and intentions among HIV positive men, we also assessed the face, content, and construct validity, as well as the test–retest reliability and internal consistency of the study instrument.

A survey instrument that can be used to assess the fertility desires and intentions of men living with HIV was developed. Content validity supported assessment as to whether the content was relevant to the concept of fertility desires and intentions. Although face validity is not the most sophisticated measure of validity, it nonetheless provides important information about the clarity of the questionnaire being completed by men living with HIV. The CVI of most of the items was ≥0.78. However, qualitative information provided by experts was used to reword 2 items. Face validity was undertaken with heterosexual and nonheterosexual men living with HIV. Results from focus group discussions helped to add a pool of community and social worker–style items that respondents understood and were willing to endorse. Factor analysis supported a 1-factor solution. Two and 1 items were removed from the construct “desires” and “intentions,” respectively, in an iterative process due to poorer factor loadings or negative contributions to the overall reliability of the scale. This process resulted in a final version with 4, 7, and 4 items for “feelings,” “desires,” and “intentions,” respectively. Notably, the factor structure in the final version matched the structure proposed by Loutfy et al.^11^ The internal reliability reached the recommended level for a new tool and test–retest indicated stability of the responses to the items. Overall, the developed instrument included higher number of constructs. Future research using this instrument will examine criterion validity, the fit of the model using confirmatory factor analysis, and the proportion of men who desire or intend to become a father using the validated constructs.

There were a number of limitations that need to be taken into account when interpreting the findings of the current study. Although the recruitment of most participants from a large urban clinic expedited completion of this study, this strategy likely introduced sampling and selection bias, as these men may differ from other men in Toronto, Ontario. Other important factors that may introduce sampling and selection bias to this pilot study include mode of HIV acquisition, socioeconomic status, and access to health care services. Because this study was conducted in an HIV clinic, it likely captured individuals who were more adherent to clinic appointments and likely more adherent to antiretroviral treatment, which may have also contributed to study bias. In addition, we had a relatively small sample size.

Another possible variable that introduced bias or confounding variables into the study involves the use of the CAPI model system. Participants may feel uncomfortable and may withhold personal and sensitive information during the interview. Questions regarding HIV acquisition and sexual history may be particularly sensitive to some participants, and this may also contribute to recall bias. Additionally, questions regarding actions taken in the last 12 months are also subject to recall bias. Although CAPI offers many advantages, a risk of possible interviewer bias will always exist. Furthermore, not all of the items identified as relating to fertility desires and intentions can directly measure the intended outcome. They were identified as important because they represent proxies for other unmeasured variables. In order to obtain quality data on all intended indicators, further research is suggested to

1. Recruit men from across Ontario.
2. Generate a sample that aligns with distribution of men in terms of geography, mode of HIV acquisition, socioeconomic status, and access to health care services.

We have developed an instrument to collect information concerning the fertility desires and intentions of men living with HIV in Ontario, Canada. This instrument will help to further our knowledge and to fill a current void in this field of research and resource allocation.

## Conclusion

Our findings demonstrate that the developed survey instrument is reliable and valid; therefore, it can be used to measure fertility desires and intentions for men living with HIV in Ontario and in other similar regions. Refinements of the instrument were recommended from the content and face validations. A pilot study using a small number of participants including heterosexual and nonheterosexual men revealed that this tool possesses internal consistency and elevated test–retest reliability. Additionally, we found that predetermined constructs can function well as single structure.

Survey development requires careful construction of instruments to ensure valid and reliable results. Having a validated comprehensive survey tool for examining various aspects of fertility desires and intentions, as well as experiences with fertility clinics, provides valuable data to policy-makers and decision-makers. This study represents the initial phase of generating evidence-based research that supports men and couples in family planning in Canada and in other countries. Researchers and clinicians will be able to use this tool to improve their understanding of the trends in fertility and issues facing men living with HIV. See Supplemental Material for the final version of the survey instrument.

## Supplemental Material

Supplemental Material, Table_S1._Final_Version_Survey - Understanding the Fertility Desires and Intentions among HIV-Positive Men Living in Ontario: Survey Instrument Development and Validation Click here for additional data file. Supplemental Material, Table_S1._Final_Version_Survey for Understanding the Fertility Desires and Intentions among HIV-Positive Men Living in Ontario: Survey Instrument Development and Validation by Joseph Nguemo Djiometio, Tony Antoniou, Vicki Kristman, Rebecca Schiff, Molly Gamble, Logan Kennedy, Mark Yudin, and Mona Loutfy in Journal of the International Association of Providers of AIDS Care (JIAPAC)
