# Supplementary material for: Understanding the Fertility Desires and Intentions among HIV-Positive Men Living in Ontario: Survey Instrument Development and Validation
Source: J Int Assoc Provid AIDS Care. 2019 Feb 25;18:2325958219831018. doi: 10.1177/2325958219831018 (PMC6748483; doi:10.1177/2325958219831018)
Supplement: Supplemental Material, Table_S1._Final_Version_Survey - Understanding the Fertility Desires and Intentions among HIV-Positive Men Living in Ontario: Survey Instrument Development and Validation [file Table_S1._Final_Version_Survey.pdf]

Table S1. Final Version Survey

Questionnaire

This document is confidential and anonymous.

Do not write your name anywhere on this document.

Please answer each question to the best of your abilities. It is important that you **provide the answer most reflective of your intentions or desires** in this questionnaire.

The staff research assistant will assist you by reading out the questions and answers for you to choose.

Your participation is optional. We understand that some of these questions may be difficult to answer. **You do not have to answer any questions and you can skip any, as needed.** We appreciate you taking the time to fill out this questionnaire.

FERTILITY DESIRES AND INTENTIONS IN MEN LIVING WITH HIV IN ONTARIO STUDY

Survey ID:

Have you filled out this questionnaire before? (Please check the box that applies)

Yes
No

Section A:Demographics

Here are some basic questions about YOU. Please do not write your name on this form

1

- a) Today's Date ______________
- b) Age (in years) ______________
- c) In what country were you born? ________________________
- d) How long have you lived in Canada? (years)________________________
- e) In what city do you currently live? _______________________________

2

Ethnic Background (check as many as apply to you):

- Caucasian
- Black
- Hispanic
- East Asian
- South Asian
- Middle Eastern
- First Nation/Métis/Inuit
- Other; Specify: ______________________
- I don't know
- Prefer not to answer

3

In the previous question, there was a list of ethnic backgrounds. However, this list may or may not specify how you identify. Regardless of your answer to the previous question, how do you identify your ethnic background (s)?

Ethnically, I identify as: ____

4

How would you describe your sexual orientation (check all that apply)?

- Straight
- Gay

- Bisexual
- Queer
- Questioning
- Two-spirit
- Other sexual orientation; Specify: ______________________

5

What gender do you identify as?

- Cisgender male
- Transmale
- Two-spirit
- Queer gender
- Other; Specify: ______________________
- I don’t know
- Prefer not to answer

***Cisgender male is** someone whose gender corresponds to their assigned sex

6

School / Work: Please check all that apply to you:

- Full time work
- Full time in school
- Part time work
- Part time in school
- Self employment
- On government assistance
- Retired
- Other; Specify: ______________________
- I don’t know
- Prefer not to answer

7

Please check the appropriate box or boxes for the following questions about religion (drop down menu)

- Jewish
- Catholic
- Protestant
- Baptist
- Evangelical
- Born -Again
- *Jehovah's Witness*
- Muslim
- Hindu
- Sikh

- Buddhist
- Eastern Orthodox
- African Traditional
- Aboriginal Traditional
- New Age
- Agnostic
- None
- Atheist
- Other
- I don’t know
- Prefer not to answer

8

a) Are you currently in a romantic and/or sexual relationship?

- Yes
- No

b) If you are currently in a relationship, have you had the same romantic partner over the past 12 months?

- Yes
- No

c) Are you currently in a monogamous romantic (single-partner) relationship?

- Yes
- No

9

What is your marital status (check as many as apply):

- Married
- Common-law partner
- Living with a partner (but not married or in a common-law relationship)
- Divorced
- Widowed
- Never Married
- Single

10

What is the highest level of education you completed?

- Did not attend high school
- Some high school education
- High school diploma
- Some university, college or technical school education
- College diploma or technical certificate
- Bachelor’s degree

- Some graduate or professional school
- Graduated graduate or professional school

11

a) Please indicate your yearly household income (as an individual).

- $0-19,999
- $20,000 - 39,999
- $40,000 - 59,999
- $60,000 - 79,999
- $80,000 - 99,999
- $100,000 - 119,999
- $120,000 or more
- I do not know
- Prefer not to answer

b) How many people in addition to you live on this household income?
0 1 2 3 4 5 6 7 8 9 or more

C) Who do you live with?

- Family
- Friend (s)
- Partner
- Spouse
- I live alone
- Others

Section B:Contraception and Sexual History

The following questions ask you about your SEXUAL HISTORY. If you do not feel comfortable answering these questions with the interviewer, you can answer them on your own.

12

a) Have you had sexual intercourse (vaginal) with a woman EVER in your lifetime? (**skip pattern**)

- Yes
- No

b) Have you had sexual intercourse (vaginal) with a woman in the last 12 MONTHS?

- Yes
- No

13

a) What is your current relationship status?

- I am single.
- I do not have a partner
- I have one primary partner (**skip pattern**)
- I have more than one partner

b) If you have a primary partner how long have you been with your partner?
(mm/yy) ___________________

c) What is your partner's status?

- HIV-negative
- HIV-positive, not on antiretroviral therapy
- HIV-positive, on antiretroviral therapy
- HIV-positive, don't know if on antiretroviral therapy
- I do not know
- I prefer to not answer

The following questions ask you about your use of BIRTH CONTROL. If you do not feel comfortable answering any of these questions with the interviewer, you can answer them on your own. (Skip pattern to be inserted: FOR – Yes to contact with female in 12 months?

14

a) Have you had a female partner in the last 12 months? (**skip pattern**)

- Yes
- No

b) Do you CURRENTLY use a method of birth control when intimate with a female partner?

- Yes
- No

c) Which methods of birth control have you or your female partner used in the past six months? (Check all that apply)

• None<br>• Male condoms for birth control<br>• Female condoms<br>• the "pill" (oral contraceptive)<br>• Diaphragm<br>• Intrauterine Devices "IUD"<br>• Injection (Depo-Provera)<br>• "Morning after pill"<br>• Rhythm method	• Implant (Norplant)<br>• Contraceptive cream<br>• Contraceptive patch<br>• Contraceptive vaginal ring<br>• Contraceptive sponge or foam<br>• My partner has had her "tubes tied" (tubal ligation)<br>• I have had a vasectomy (male sterilization)<br>• I do not have sex with women<br>• Other

• Withdrawal	

Section C:Interest/Desire to Have Children

The following questions ask you about your FEELINGS (Feelings are emotions).

15

	Strongly Disagree	Disagree	Neither disagree nor agree	Agree	Strongly Agree
• I think being a father is important to me.					
• I think I would feel fulfilled by caring for children.					
• I think that being a father would increase my worth in life.					
• I think children give meaning to life.					

The following questions ask you about your DESIRES (Desires is the want for something)

16

	Strongly Disagree	Disagree	Neither disagree nor agree	Agree	Strongly Agree
• I have thought about becoming a father in the future.					
• I would like to become a father in the future.					
• I would like to become a father without parenting the child(ren).					
• Being diagnosed with HIV affects my decisions about becoming a father.					
• Being diagnosed with HIV affects my decisions about not becoming a father.					
• If I was in a situation where I could become a father, I would want that to happen.					
• Available fertility technologies and options for people living with HIV affect my decisions about becoming a father.					

17

a) How many children would you like to parent in the future?
0 1 2 3 4 5 6 7 8 or more

b) What would be the best timeframe for you to become a father?

- Never
- Now
- Within 6 months
- Within 1 year
- Within 2 years
- Within 3 years
- Within 4 years
- Within 10 years
- I do not know
- I prefer to not answer

Section D: Intent to Have Children in the Future

***Intention is what we are going to do**

The following questions ask you about your PLANS to have children.

18

a) How many children do you expect to parent in the future?
0 1 2 3 4 5 6 7 8 or more

b) How soon in the future do you plan to become a parent?

- Never
- I am expecting a child now/awaiting adoption
- Within 6 months
- Within 1 year
- Within 2 years
- Within 3 years
- Within 4 years
- Within 10years
- I do not know
- I prefer to not answer

19

	Strongly Disagree	Disagree	Neither disagree nor agree	Agree	Strongly Agree
• I am open to the idea of using medical techniques to help me become a father [such as in vitro fertilization (IVF) or intra-uterine insemination (IUI).					
• Because HIV medications will let me live longer, I am considering becoming a father.					
• As a man with HIV, I can have an HIV-negative child.					
• I am willing to pay for fertility clinic resources to becoming a father.					

The following questions ask you about your WORRIES.

20

If I were to become a father	Strongly Disagree	Disagree	Neither disagree nor agree	Agree	Strongly Agree
• I would be worried that I will not have enough help from friends or family to care for children in the future.					
• I would be worried that being a father will affect my health.					
• I would be worried that my child will be born HIV positive.					
• I would be worried that I will not be healthy enough to care for children into adulthood.					
• I would be worried that my HIV medications will affect my ability to care for my child					
• I would be worried that if I have children I would not focus enough on my medication					
• I would be worried that I will not live long enough to care for children into adulthood.					
• I would be worried about infecting the woman who carried the pregnancy (regardless of my relationship with her).					
• I would be worried that my children will experience discrimination at school.					

• I would be worried that I will not have enough money to care for children.					
• I would be worried that I don't feel mature enough to be become a father.					

Section E:Behaviour(s) Related to the Pursuit of Fertility

The following questions ask you about your ACTIONS in the last 12 months,

21

	YES NO N/A
• I have approached my partner/spouse/friend/other about having a baby (other = significant other, friend or other person that a man would consider having a child with)	
• I have spoken to my birth mother or co-parent about having a baby	
• I have spoken to a community member about having a baby	
• My partner/spouse/friend/other has approached me about having a baby.	
• I have spoken to my doctor, about becoming a father.	
• I have spoken to my nurse, about becoming a father.	
• I have spoken to my nurse practitioners about becoming a father.	
• I have spoken to other healthcare professionals about becoming a father.	
• I don't take actions to avoid having children	
• I went to sessions about adoption	
• I filled out application forms with adoption organizations	
• I looked into organizations that broker surrogacy agreements	

22

a) Have you searched for information on fatherhood in the past 12 months?
- Yes
- No

b) If Yes, where have you searched for information? Check all that apply.
- Internet
- Magazines
- Newspapers
- AIDS Service Organization (such as your local AIDS Committee)
- Community Health Centre

- Family doctor
- HIV specialist
- Obstetrician / Gynaecologist (OB/GYN)
- Nurses
- Nurse practitioners
- Other HIV-positive fathers
- Networks
- Talking to friends/acquaintances
- Other

Section F:Sexual intercourse and Conception/Parenting History

23

a) How would you describe your behaviour at the present time when you have sexual intercourse with women?

- I do not have sex with women
- I use some kind of birth control every time I have sex to avoid pregnancy
- I use some kind of birth control when I think there is a risk of pregnancy
- I don't use any kind of birth control and I wouldn't be unhappy about pregnancy
- I don't use any kind of birth control but I am not trying to achieve a pregnancy
- I don't use any kind of birth control and I am trying to achieve a pregnancy

The following questions ask you about your experience with CONCEPTION/PARENTING.

24

a) How many children have you ever parented?

0 1 2 3 4 5 6 7 8 or more

b) How many children have you ever parented since you were diagnosed with HIV?

0 1 2 3 4 5 6 7 8 or more

c) How many times have you EVER gotten a woman pregnant? (Include abortions and miscarriages)
0 1 2 3 4 5 6 7 8 or more

d) How many of these pregnancies were planned:_____

e) How many times have you EVER gotten a woman pregnant SINCE you were diagnosed with HIV? (Include abortions and miscarriages)
0 1 2 3 4 5 6 7 8 or more

f) How many of these pregnancies were planned:_____

Section G:Perceived Support for Becoming a Parent

25
a)

	Strongly Disagree	Disagree	Neither disagree nor agree	Agree	Strongly Agree
• My family wants me to be a father					
• By having a child, it would make my family happy					

b)

	Strongly Disagree	Disagree	Neither disagree nor agree	Agree	Strongly Agree
• My community wants me to have a child					
• By having a child, it would make my community happy.					

C (skip pattern)

	Strongly Disagree	Disagree	Neither disagree nor agree	Agree	Strongly Agree
• My partner wants me to be a parent in the future.					
• If we have a child, it would make my partner happy.					

26

The following questions ask you about your experience with FEAR. If you do not feel comfortable answering any of these questions, skip to the next one.

27

	Definite False	Somew false	Neither true nor false	Somewha true	Definitely true
• I am afraid of being judged negatively By a friend for trying to become a father					
• I am afraid of being judged negatively By a family member for trying to become a father					
• I am afraid of being judged negatively by my other child (ren) for trying to become a father					
• I am afraid of being judged negatively By a physician for trying to become a father					
• I am afraid of being judged negatively By another health care professional for trying to become a father					
•					
• I am confident I would be able to meet other HIV+ parents to share experiences with if I had children of my own					

The following questions ask you about your EXPERIENCES. If you do not feel comfortable answering any of these questions with the interviewer, you can answer them on your own.

28

	Definitely False	Somewhat False	Neither True nor False	Somewhat True	Definitely True
• I have been judged negatively by a friend for trying to become a father					
• I have been judged negatively by a family member for try to become a father					
• I have been judged negatively by a physician for trying to become a father					
• I have been judged negatively by another health care					

professional for trying to become a father					
• I have not been Judged negatively by anyone for trying become a father					

Section H: Satisfaction with Providers related to Fertility Goals

The following questions ask you about your health care providers.

29

a) Do you have a family doctor?

- Yes
- No

b) If yes, please complete the questions below. If you do not have a family doctor, please skip this set of questions

	Definitely False	Somewhat False	Neither true nor false	Somewhat true	Definitely true
• I am comfortable sharing my concerns about becoming a father with my family doctor.					
• I am comfortable talking to my family doctor about fatherhood as a man living with HIV.					
• I can trust my family doctor.					
I am satisfied with the treatment I receive from my family doctor.					
• I am satisfied with the amount of parenting planning information I received from my family doctor.					

c) Has your family doctor talked to you about fatherhood?

- Yes
- No
- I don't know
- I prefer not to answer

d) Has your health family doctor ever advised you against having children?

- Yes
- No
- I don't know
- Prefer not to answer

30

a) Do you see an HIV Specialist?

- Yes, Same doctor as my family doctor
- Yes, Different doctor than my family doctor
- Yes, Different doctor than my family doctor and also my family doctor – both are my HIV Specialists
- No

b) If yes, please complete the questions below. If you do not see an HIV Specialist, please skip this section

	Definitely False	Somewhat False	Neither true nor false	Somewhat true	Definitel[illegible] true
• I am comfortable sharing my concerns about becoming a father with my HIV Specialist.					
• I am comfortable talking to my HIV Specialist about fatherhood as a man living with HIV.					
• I can trust my HIV Specialist.					
• I am satisfied with the treatment I receive from my HIV Specialist.					
• I am satisfied with the amount of parenting planning information I received from my HIV Specialist.					

c) Has your HIV Specialist talked to you about fatherhood?

- Yes
- No
- I don't know
- Prefer not to answer

d) Has your specialist ever advised you against having children?

- Yes
- No
- I don't know
- Prefer not to answer

31

a) Do you see case /social worker?

- Yes
- No

b) If yes, please complete the questions below. If you do not see a case worker/social worker, please skip this section (skip pattern)

	Definitel[illegible]	Somewh[illegible]	Neither true	Somewhat	Definitel[illegible]

	False	False	nor false	true	true
• I am comfortable sharing my concerns about becoming a father with my case /social worker					
• I am comfortable talking to my case /social worker about fatherh[illegible] a man living with HIV.					
• I can trust my case /social<br>• worker					
• I am satisfied with the service I receive from my case /social worker.					
• I am satisfied with the amount of parenting planning information I received from my case /social worker					

c) Has your case /social worker talked to you about fatherhood?

- Yes
- No
- I don't know
- I prefer not to answer

d) Has your case /social worker ever advised you against having children?

- Yes
- No
- I don't know
- I prefer not to answer

32

c) Do you see nurses, nurse practitioners or other health care providers?

- Yes
- No

d) If yes, please complete the questions below. If you do not see a case worker/social worker, please skip this section (skip pattern)

e) If yes, please complete the questions below. If you do not see a case worker/social worker, please skip this section (skip pattern)

	Definitely False	Somewhat False	Neither true nor false	Somewhat true	Definitely true
• I am comfortable sharing my					

concerns about becoming a father with my nurse or nurse practitioner or other health care providers.					
• I am comfortable talking to my family nurse or nurse practitioner other health care providers about fatherhood as a man living with HIV.					
• I can trust my nurse or nurse practitioner or other health care providers.					
I am satisfied with the care I receive from my nurse or nurse practitioner or other health care providers.					
• I am satisfied with the amount of parenting planning information I received from my nurse or nurse practitioner or other health care providers.					

f) Has your nurse, or nurse practitioner or other health care providers talked to you about fatherhood?

- Yes
- No
- I don't know
- I prefer not to answer

g) Has your nurse, or nurse practitioner or other health care providers talked to you about fatherhood?

- Yes
- No
- I don't know
- I prefer not to answer

Section I:Needs Assessment

33

Select which resources would help you with your decisions to become a father? (Check all that apply)

- I would need information booklets and publications on becoming a father
- I would need to talk to an HIV-positive man who is already a parent
- I would need to talk to a health professional with fertility expertise

- I would need to talk someone with fertility expertise from my community
- I would need access to a fertility clinic
- I would need an obstetrician
- I would need a trained midwife
- I would need to talk to a social worker
- I would need access to egg banks/surrogates
- I would need educational seminars on conception
- I would need educational seminars on taking care of a baby
- I would need educational seminars on raising a child
- I need courses in adoption
- I need to talk to adoption organizations
- I need to talk to organizations that broker surrogacy agreements
- Other

Section J: HIV History

The following questions ask you about your HIV infection. If you do not feel comfortable answering any of these questions, skip to the next one.

34

In what year did you test HIV positive?

35

How do you think you got HIV? (Check all that apply)

- Sex with a male partner
- Sex with a female partner
- Sexual assault (rape)
- Needle sharing with infected person
- Blood transfusion / blood product
- From your birth mother
- Occupational exposure / needle stick injury
- Other; Specify: ___________________
- I don't know
- Prefer not to answer

36

Please tell us the LAST TIME you were told your CD4 cell count, and what your CD4 cell count was at that time. (CD4 cells are also known as "T cells" or your "immune cells", and they are usually between 0 and 1000).

a)Last time you were told your CD4 (mm/yyyy):

b) CD4 cell count. Please write the number here:

c) I don't remember / I don't know

d) I have never had a CD4 test

e) I prefer not to answer

37

Please tell us the LAST TIME you were told your viral load, and what your viral load was at that time. (Usually, viral loads range from undetectable to 100,000+)

a)Last time you were told your viral load (mm/yyyy)

b)Viral load: **or** UNDETECTABLE (<50)

c) I don't remember / I don't know

d) I have never had a viral load test

e) I prefer not to answer

38

a) Have you EVER taken HIV medications?

- Yes
- No

e) IfYes, when did you start taking HIV medications (mm/yyyy)?

39

a) Are you currently taking HIV medications?

- Yes
- No

b) If yes do have concerns that the HIV medications you take could affect your child?

- Yes
- No

40

Do you currently have any of the following sexually transmitted infections? (Check all that apply)

a)

- Hepatitis B
- Chlamydia
- Gonorrhea
- Syphilis
- HPV (genital warts)
- Herpes (genital)
- Never been diagnosed
- None
- I do not know
- I prefer not to answer

b)Hepatitis C (skip pattern)

Yes

No

c) If yes, are you currently taking hepatitis C treatment? (This medication should not be taken when trying to have a child)

- Yes
- No
- I don’t know
- Prefer not to answer

d) If YES, have any of your doctors told you that Hepatitis C treatment is not recommended for people trying to conceive?

- Yes
- No
- I don’t know
- Prefer not to answer
